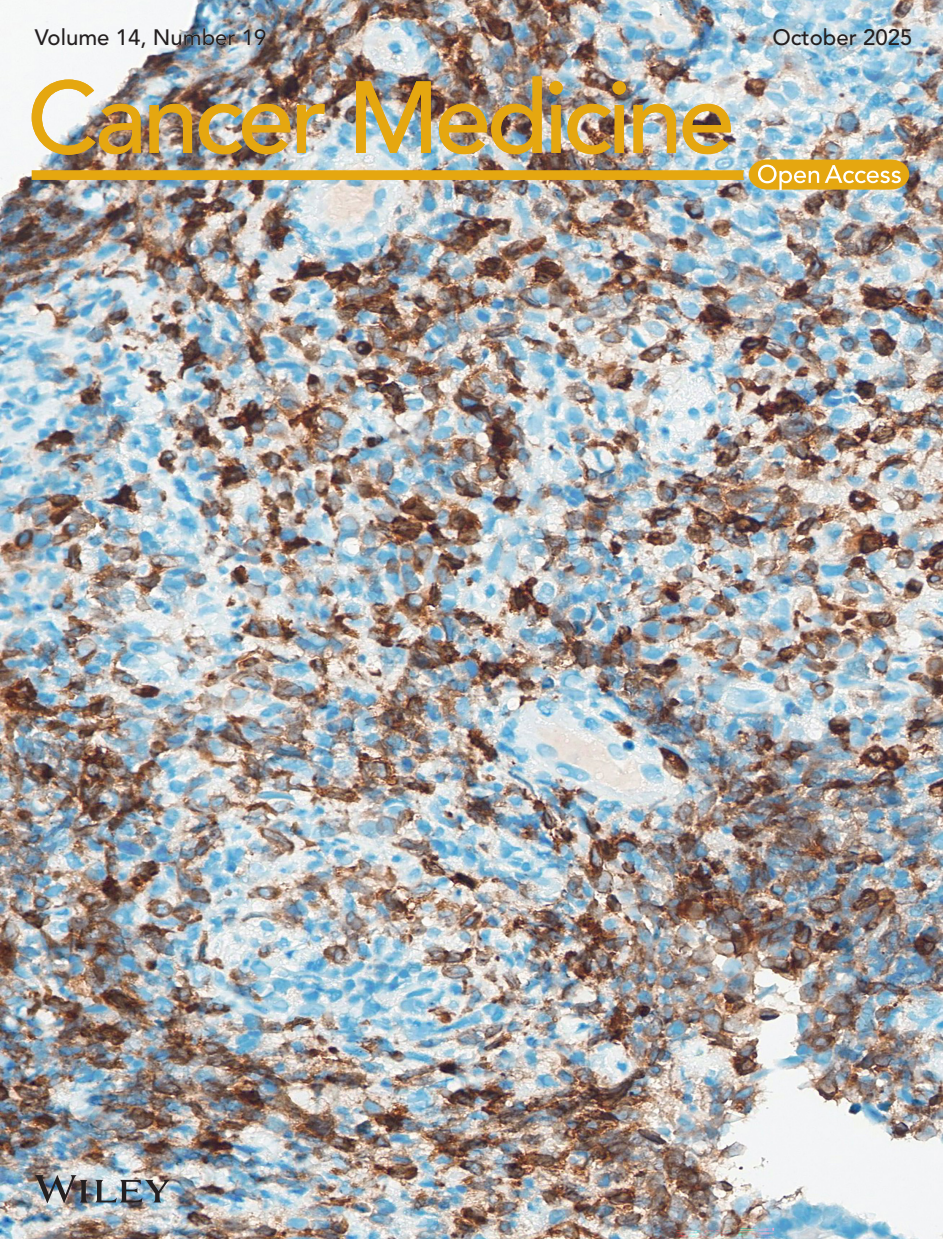# Cover Image

**DOI:** 10.1002/cam4.71384

**Published:** 2025-11-17

**Authors:** Mbwamulungu Nakweti Julia, Azako David, Pezo Serge, Bokambadja Fabrice, Kapour Kieng Germain, Bompangue Nkoko Didier, Kisile Olive, Lebwaze Massamba Bienvenu, Kabongo Mpolesha Jean‐Marie

## Abstract

The cover image is based on the article *Epidemiological Profile of Non‐Hodgkin's Lymphomas Seen at Kinshasa University Clinics From 2012 to 2022* by Mbwamulungu Nakweti Julia et al., https://doi.org/10.1002/cam4.71254.